# Analysis of Factors Associated With Depression in Community-Dwelling Older Adults in Wuhan, China

**DOI:** 10.3389/fnagi.2021.743193

**Published:** 2021-11-12

**Authors:** Aihong Liu, Yingjie Peng, Wenli Zhu, Yanling Zhang, Shihui Ge, Yun Zhou, Kemeng Zhang, Zhaohui Wang, Ping He

**Affiliations:** Department of Geriatrics, Union Hospital, Tongji Medical College, Huazhong University of Science and Technology, Wuhan, China

**Keywords:** community, older adults, depression, frailty, social support, cognitive dysfunction

## Abstract

**Objectives:** This study aimed to identify the independent factors associated with depression in community-dwelling older adults in Wuhan, China.

**Methods:** Four hundred and seventy older adults (aged ≥65 years) from four communities dwelling on Junshan Street in Wuhan, China were included in this study. Participants completed a questionnaire that asked questions pertaining to age, gender, educational level, income, living situation, care situation, social support, and social engagement. The 30-item Geriatric Depression Scale (GDS-30), the Fried frailty phenotype scale, the activities of daily living (ADL) scale, the mini nutritional assessment scale-short form (MNA-SF), and the Mini-cog scale were used to assess depression, frailty, self-care ability, malnutritional risk, and cognitive dysfunction, respectively. Differences in age, gender, educational level, income, living situation, care situation, social support, social engagement, ADL score, risk of malnutrition, frailty, and cognitive dysfunction between the non-depression (GDS-30 score <10 points) and depression groups (GDS-30 score ≥10 points) were compared using a chi-square test. Moreover, correlations between factors and depression were analyzed using Pearson’s correlation. Then, significant variables (*p* < 0.05) from the chi-square test were included in a multivariable logistic regression model to identify the independent factors associated with depression.

**Results:** The incidence of depression among the participants was 14.04%. Age (*p* < 0.001), educational level (*p* < 0.001), living situation (*p* < 0.001), social support (*p* = 0.001), ADL score (*p* = 0.023), frailty (*p* < 0.001), and cognitive dysfunction (*p* < 0.001) were all significantly associated with depression, in which age, poor social support, frailty, and cognitive dysfunction were identified as independent factors.

**Conclusion:** Improving social support and effective interventions for frailty and cognitive dysfunction may help relieve depression in community-dwelling older adults.

## Introduction

Depression is considered a disease of modernity, as modernization is generally associated with higher rates of depression (Hidaka, 2012). With the increase in life expectancy and an increase in the older adult population worldwide, medical, psychological, and social problems among older adults have become a major concern (World Health Organization (WHO), 2011). Depression is a commonplace vulnerability in older adults. It may lead to social impairment, decreased quality of life (McCall and Kintziger, 2013), negative impact on chronic diseases, and even increased risk of suicide (Kaneko et al., 2007). Depression causes considerable suffering and leads to impaired functioning in daily life. Among those aged over 60 years, depression is both underdiagnosed and undertreated in primary care settings. Symptoms are often overlooked and untreated because they usually co-occur with other problems encountered by older adults (World Health Organization, 2017). Moreover, a low mood state in the elderly is often misinterpreted as an aging phenotype (Segel-Karpas et al., 2017). Therefore, identifying geriatric depression and its associated risk factors among the elderly is crucial. In this study, older adults from four communities in Wuhan were studied to understand the prevalence of depressive symptoms in the community and to analyze the factors involved in improving care for geriatric depression. In the field of public health, this may also aid in the ultimate prevention of geriatric depression.

## Materials and Methods

### Study Design and Participants

Of the 21 communities in Junshan Street, which is located in the urban–rural area of Wuhan City, four communities were selected for this study using random cluster sampling; the study period was from September 25, 2020 to October 31, 2020. The minimum sample size was 463 (α = 0.05, δ = 0.03, and *P* = 0.85). Ultimately, 470 individuals were included in this study. The following were the inclusion criteria: (1) age ≥65 years; (2) permanent residents of the community, having lived in the community for over 6 months; and (3) able to provide informed consent, communicate well, and complete a questionnaire and physical assessments. The following were the exclusion criteria: (1) those with severe mental illnesses, such as schizophrenia and bipolar disorder; (2) those who had a critical or terminal illness, were completely disabled, or were bedridden; and (3) those with severe hearing, vision, and/or language impairments that prevented them from cooperating or completing the assessments. All subjects provided informed consent. The study protocol was approved by the ethics committee of Union Hospital, Tongji Medical College, Huazhong University of Science and Technology.

The investigators of this study conducted the questionnaires survey and physical assessments such as muscle strength and gait velocity of the participants using standardized protocols.

### General Information Survey

A questionare survey was used to collect data such as the participants’ age, gender, educational level, income, living situation, care situation, social support, and social engagement. The income means monthly income. Compared with the local minimum living expenses, the income situation is divided in three groups: with surplus after expenses, just enough to cover expenses, and not enough to cover expenses. The living situation includes living alone and living with spouse or relatives. The care situation includes care for oneself and care by spouse or relatives. Social support includes material and emotional support from others. In terms of strength of social support, it was divided into three categories: sufficient material and emotional support, only material or only emotional support available, and lack of any material or emotional support. Furthermore, social engagement includes participating in family occasions, community activities, or religious activities. Attending social engagements once a week or more was defined as proactive and active participation. Attending social engagements less than once a week and once a month or more was defined as “regular participation.” Attending social engagements less than once a month and once in half a year or more was defined as “occasional participation.” Finally, attending social engagements less than once in half a year was defined as “no participation.”

### Assessment of Depression

The Geriatric Depression Scale (GDS-30) (Yesavage et al., 1982; Krishnamoorthy et al., 2019) was used to assess whether or not the participants had depression. The total score on this scale ranges from 0 to 30 points. A score of 11–20 points indicates mild depression, and a score of 21–30 points indicates moderate to severe depression.

### Activities of Daily Living Scale

The activities of daily living (ADL) function among older adults was identified using two scales (Lawton and Brody, 1969): (1) the physical self-maintenance scale (PSMS), which has six items: ability to go to the toilet, feeding, dressing, grooming, ambulation, and bathing; and (2) the instrumental activities of daily living (IADL) scale, which consists of eight items: ability to use a telephone, shopping, food preparation, housekeeping, laundry, mode of transportation, responsibility for own medications, and ability to handle finances. A total ADL score <16 is considered normal, whereas a score ≥16 indicates dysfunction. If a subject scores ≥3 for two or more items or has a total score ≥22, they are considered to have significant dysfunction.

### Mini Nutritional Assessment-Short Form

The mini nutritional assessment-short form (MNA-SF) consists of six items: decline in food intake, weight loss, mobility, psychological stress or acute disease, neuropsychological problems, and body mass index (BMI). The scale is scored out of a total of 14 points, with a score of 11–14 indicating no risk of nutrition and a score of <11 points indicating risk of malnutrition.

### Fried Frailty Phenotype Assessment

The Fried frailty phenotype scale comprises five indicators: (1) weight loss >3 kg or >5% in 1 year; (2) perceived exertion: feeling exhausted or lacking energy for more than 3 days in the previous week; (3) decreased muscle strength: handgrip strength (men ≤23 kg; women ≤14 kg); (4) decreased physical function: gait velocity (15 feet or 4.5 meters) men ≥7 s, women ≥7 s; and (5) decreased somatic exercises: men <383 kcal/week (approximately 2.5 h of walking), women <270 kcal/week (approximately 2 h of walking). One point was given for each indicator. If none of the criteria are fulfilled, zero points are given, and the patient is considered non-frail. If 1–2 indicators of the criteria are fulfilled, 1–2 points are given, and the patient is classified as pre-frail. If 3–5 indicators of the criteria are fulfilled, 3–5 points are given, and the patient is classified as frail.

### Cognitive Dysfunction Assessment

The Mini-cog scale consists of two simple cognitive tests, which include a three-item memory-recall test and a clock-drawing test (CDT) (Borson et al., 2000; McCarten et al., 2011; Chan et al., 2019). In the memory-recall test, the participant is asked to listen carefully and remember three unrelated words and recall them. In the CDT, the participant is asked to draw the shape of a clock on a blank piece of paper, mark the scale, and mark a selected moment on the clock within a specified time. For the memory-recall test, one point is awarded for each of the three words correctly recalled. In this study, participants were divided into two groups: those with cognitive dysfunction (Yes) and those without (No) cognitive dysfunction. Participants without cognitive dysfunction scored a total of three points in the memory-recall tests or two points in the memory-recall tests with correct CDT. Those with cognitive dysfunction scored 0–1 points in the memory-recall tests or two points in the memory-recall tests with incorrect CDT.

### Statistical Analyses

Categorical variables are expressed as numbers and percentages (%). The differences between the depression and non-depression groups were analyzed using a chi-square test. Furthermore, correlations between the factors and depression were analyzed using Pearson’s correlation. Significant variables (*p* < 0.05) from the chi-square test were included in a multivariable logistic regression model to identify the independent factors that contributed to depression. *P-*values <0.05 were considered statistically significant. Data were analyzed using the SPSS Statistics software (version 24.0, IBM, Armonk, NY, United States).

## Results

### Variables Associated With Depression in Community-Dwelling Older Adults

None of the participants in this study had moderate or severe depression. The scores of depressed participants were in the range of 10–20 points. The 470 participants were divided into two groups according to the GDS-30 assessment: non-depression (GDS-30 score <10 points) and depression (GDS-30 score ≥10 points) groups. The ages of the participants ranged from 65 to 92 years. The depression group had 66 participants (14.04%). Moreover, the differences of depression morbidity in the age groups of 65–75 years, 75–85 years, and ≥85 years were significant (*p* < 0.001). In addition, the prevalence of depression was found to increase with age (*r* = 0.395, *p* < 0.01). Men constituted 41.91% of the sample, and there were no significant differences in the prevalence of depression between genders. Furthermore, 186 participants were illiterate, 115 were primary school graduates, 106 were middle school graduates, and 63 were high school graduates; none of the subjects had a college degree or higher. The difference in the level of education was significant between the non-depression and depression groups (*p* < 0.001), with a higher level of education being associated with the lower prevalence of depression (*r* = −0.300, *p* < 0.01). For 8.09% of the sample, monthly income was not enough to cover expenses, 54.47% of participants had monthly income to cover expenses only, and 37.45% of people had surplus income each month. The income conditions of those in the depression and non-depression groups did not differ significantly (*p* = 0.258). Furthermore, the living situation differed significantly between the non-depression and depression groups (*p* < 0.001); 17.45% of the older adults lived alone; living alone was significantly associated with depression (*r* = −0.169, *p* < 0.01), and 75.53% of participants reported self-care. The prevalence of depression between those who self-cared and those who were cared for by a spouse or relatives did not differ significantly (*p* = 0.200).

With regard to material and emotional support, 54.04% of participants reported they received sufficient support. The prevalence of depression significantly differed among different social support conditions (*p* = 0.001), with better social support being associated with a lower prevalence of depression (*r* = 0.164, *p* < 0.01). Only 10.85% of the participants actively participated in social engagements, 9.79% of the participants participated regularly in social engagements, 30.43% of the participants participated occasionally in social engagements, and 48.94% never participated in social engagements. However, participation in social engagements did not differ significantly between the depression and non-depression groups (*p* = 0.147). Moreover, ADL was considered normal in 406 older adults; 48 had dysfunctional ADL, and 16 had significant ADL dysfunction. The ADL condition differed significantly between the depression and non-depression groups (*p* = 0.023). Furthermore, according to the MNA-SF scale, 24.47% of older adults in this study were at risk of malnutrition. However, the risk of malnutrition did not significantly differ between the depression and non-depression groups (*p* = 0.134). In addition, non-frailty was observed in 58.09% of older adults, 35.32% had pre-frailty, and 6.60% had significant frailty. The level of frailty differed significantly between the depression and non-depression groups (*p* < 0.001) and was positively correlated with depression (*r* = 0.327, *p* < 0.01). In addition, cognitive dysfunction was identified in 39.40% of the participants, and 55.20% of those in the depression group had cognitive dysfunction (vs. 36.70% in non-depression group, *p* < 0.001). It demonstrated that the presence of cognitive dysfunction indicated a higher vulnerability to depression (*r* = 0.132, *p* < 0.01). The results are summarized in Table 1.

**TABLE 1:** Variables associated with depression in community-dwelling older adults in Wuhan, China.

	Non-depression n (%)	Depression n (%)	Total, n (%)	χ^2^	*P*	*r*
**Age (years)**				73.59	<0.001	0.395**
65–75	358 (88.61)	31 (46.97)	389 (82.77)			
75–85	43 (10.64)	29 (43.94)	72 (15.32)			
≥85	3 (0.74)	6 (9.09)	9 (1.91)			
**Gender**				0.032	0.858	0.008
Male	170 (42.08)	27 (40.91)	197 (41.91)			
Female	234 (57.92)	39 (59.09)	273 (58.09)			
**Educational level**				47.19	<0.001	−0.300**
Illiterate	136 (33.66)	50 (75.76)	186 (39.57)			
Primary school	102 (25.25)	13 (19.70)	115 (24.47)			
Middle school	104 (25.74)	2 (3.03)	106 (22.55)			
High school	62 (15.35)	1 (1.52)	63 (13.40)			
**Income**				2.708	0.258	−0.057
With surplus after expenses	149 (36.88)	27 (40.91)	176 (37.45)			
Just enough to cover expenses	219 (54.21)	37 (56.06)	256 (54.47)			
Not enough to cover expenses	36 (8.91)	2 (3.03)	38 (8.09)			
**Living situation**				13.46	<0.001	−0.169**
Alone	60 (14.85)	22 (33.33)	82 (17.45)			
With spouse or relatives	344 (85.15)	44 (66.67)	388 (82.55)			
**Care situation**				1.642	0.200	−0.059
Self-care	301 (74.50)	54 (81.82)	355 (75.53)			
By spouse or relatives	103 (25.50)	12 (18.18)	115 (24.47)			
**Social support**				14.02	0.001	0.164**
Enough material and emotional support	229 (56.68)	25 (37.88)	254 (54.04)			
Only material or emotional support	155 (38.37)	31 (46.97)	186 (39.57)			
Lack of material and emotional support	20 (4.95)	10 (15.15)	30 (6.38)			
**Social engagement**				5.366	0.147	0.089
Proactive and active participation	46 (11.39)	5 (7.58)	51 (10.85)			
Regular participation	41 (10.15)	5 (7.58)	46 (9.79)			
Occasional participation	128 (31.68)	15 (22.73)	143 (30.43)			
Never	189 (46.78)	41 (62.12)	230 (48.94)			
**ADL**				7.574	0.023	0.091*
Normal	352 (87.13)	54 (81.82)	406 (86.38)			
Dysfunctional	42 (10.40)	6 (9.09)	48 (10.21)			
Obvious dysfunctional	10 (2.48)	6 (9.09)	16 (3.40)			
**MNA-SF**				2.244	0.134	−0.069
Risk of malnutrition	94 (23.27)	21 (31.82)	115 (24.47)			
No risk of malnutrition	310 (76.73)	45 (68.18)	355 (75.53)			
**Frailty**				58.14	<0.001	0.327**
Non-frailty	263 (65.10)	10 (15.15)	273 (58.09)			
Pre-frailty	119 (29.46)	47 (71.21)	166 (35.32)			
Frailty	22 (5.45)	9 (13.64)	31 (6.60)			
**Cognitive dysfunction**				8.237	<0.001	0.132**
Yes	148 (36.70)	37 (55.20)	185 (39.40)			
No	255 (63.30)	30 (44.80)	285 (60.60)			

*p < 0.05, **p < 0.01 (two-tailed), significant correlation

*ADL, activities of daily living; MNA-SF, mini nutritional assessment-short form.*

### Independent Factors Associated With Depression in Community-Dwelling Older Adults

Univariable analyses revealed that age, educational level, living situation, social support, ADL dysfunction, frailty, and cognitive dysfunction were significantly associated with depression (Table 1). To identify the independent factors associated with depression in community-dwelling older adults, we conducted multivariable logistic regression analyses (Table 2). Being in the 75–85 years age group (*p* < 0.001) or over 85 years (*p* < 0.001), having no material or emotional support (*p* = 0.018), pre-frailty (*p* < 0.001) or frailty (*p* = 0.001), and cognitive dysfunction (*p* = 0.005) were significantly associated with depression. However, educational level, living situation, and ADL dysfunction were not significantly related to depression in community-dwelling older adults in Wuhan, China.

**TABLE 2:** Logistic regression models to identify factors associated with depression in community-dwelling older adults in Wuhan, China.

	B	SE	Wald	df	OR	95% CI	*P*
**Age (years)**							
65–75					1		
75–85	1.767	0.332	28.31	1	5.855^1^	3.054–11.23	<0.001
≥85	2.878	0.808	12.69	1	17.77^2^	3.649–86.55	<0.001
**Social support**							
Enough material and emotional support					1		
Only material or emotional support	0.539	0.332	2.632	1	1.715^3^	0.894–3.289	0.105
Lack of material and emotional support	1.198	0.508	5.553	1	3.314^4^	1.223–8.978	0.018
**Frailty**							
Non-frailty					1		
Pre-frailty	2.057	0.384	28.64	1	7.819^5^	3.682–16.61	<0.001
Frailty	1.917	0.553	12.02	1	6.803^6^	2.301–20.11	0.001
**Cognitive dysfunction**							
No					1		
Yes	0.754	0.267	7.998	1	2.125^7^	1.260–3.583	0.005

*^1^75–85 years vs. 65–75 years; ^2^ ≥85 years vs. 75–85 years; ^3^only material or emotional support vs. enough material and emotional support; ^4^ lack of material and emotional support vs. only material or emotional support; ^5^ pre-frailty vs. non-frailty; ^6^ frailty vs. pre-frailty; ^7^cognitive dysfuncion vs. normal cognition. 95% CI, 95% confidence interval; OR, odds ratio.*

Furthermore, the risk of depression in older adults aged 75–85 years was 5.855 times higher (95% confidence interval [CI]: 3.054–11.23, *p* < 0.001) than that in older adults aged 65–75 years. The risk of depression in older adults aged over 85 years was 17.77 times higher (95% CI: 3.649–86.55, *p* < 0.001) than that in older adults aged 75–85 years. Those lack of material or emotional support had 3.314 times higher risk of depression than those with only material or emotional support (95% CI: 1.223–8.978, *p* = 0.018). The risk of depression in pre-frailty older adults was 7.819 times higher (95% CI: 3.682–16.61, *p* < 0.001) than that in non-frailty older adults. In addition, the risk of depression in frailty older adults was 6.803 times higher (95% CI: 2.301–20.11, *p* = 0.001) than that in pre-frailty older adults. Moreover, the risk of depression in participants with cognitive dysfunction was 2.125 times higher (95% CI: 1.260-3.583, *p* = 0.005) than those with normal cognition. The results are demonstrated in Table 2.

## Discussion

This study found that the prevalence of depression among older adults in the Wuhan community was 14.04%. Age, educational level, life status, social support, ADL impairments, frailty, and cognitive dysfunction were associated with the prevalence of depression. Thus, old age, poor social support, frailty, and cognitive dysfunction were independent risk factors for developing depression in older adults.

Furthermore, the prevalence of depression in older adults in the Wuhan community was lower than that of older adults in western China, which was 19.6% (Zhao et al., 2020), and higher than that of older adults in Jinan, Shandong Province, China, which was reported to be 11.6% (Jin et al., 2020). Similar results were reported in a national cohort study of people aged over 65 years in Japan (Shimada et al., 2019). The reasons for such variability may be related to geographic characteristics, sample size, population distribution, and screening tools. Nevertheless, geriatric depression has emerged as a major public health problem worldwide. Previous studies have shown that depression in older adults is often perceived as a physical illness–such as sleep disorder, dementia, or geriatric problem–which are often considered natural and inevitable (Reynolds et al., 2019). Furthermore, a majority of older adult patients with depression are not diagnosed and treated promptly, which imposes heavy burdens on their families and the society.

In this study, we found that the prevalence of depression in older adults increased with age, which is consistent with the results of previous studies (Glaesmer et al., 2011). In addition, older adults may experience more psychosocial events, such as the death of relatives and friends and breakdown of marital relationships, which may lead to the accumulation of emotional stress with age (Chiao et al., 2009). Moreover, the number and severity of chronic illnesses increase with age, which may also increase the risk of developing depression (Jorm, 2000). Older adults who are excessively concerned about physical diseases experience a greater negative impact on their mental health, which can further induce the onset of depression. Furthermore, chronic pain–particularly when combined with social isolation, social stress, and poverty–is potently depressogenic in older adults.

Social support is defined as the support an individual receives through social connections with other individuals, groups, and the larger community (Lin et al., 1979). Similar with previous study (Simkhada et al., 2018), our analysis revealed that different social support conditions had significant effects on the prevalence of depression, where better social support was related to a lower prevalence of depression. Older adults are in a state of constant stress due to a lack of defined social roles and deteriorating health, which leads to a decline in the ability of individuals to cope with stressful events and a tendency to develop a negative mindset. Therefore, positive family relationships and adequate emotional support are conducive to maintaining the psychological health of older adults. Furthermore, older adults who are bereaved or living alone must receive specific attention to encourage participation in social recreational activities, acquire social roles, and be provided with emotional support to establish an effective social support system.

Numerous studies have found an interaction between frailty and depression in older adults (Buigues et al., 2015). In a meta-analysis conducted in the United Kingdom, the overall prevalence of depression among 8023 frail people was 38.6%, with a frailty prevalence of 40.40% among 2167 depressed people; moreover, frail older adults were four times more likely to suffer from depression than non-frail individuals (Soysal et al., 2017). This suggests that depression increases with the increase in frailty. Woods et al. (2005) noted that frailty is a geriatric syndrome that is affected by various factors, such as underweight, obesity, smoking, and depressive symptoms. The accompanying pain, weakness, and low activity tolerance may cause disability or functional dependence, which leads to sadness and helplessness, that in turn, promotes depression. Furthermore, depression may contribute to frailty because of the depression-related sedentary lifestyle, increased risk of falls, and weight loss, all of which increase the risk of frailty (Lohman et al., 2015). Supplementing the diet with protein and vitamin D and engaging in strengthening exercises—including impedance exercise and aerobic exercise–reduce the occurrence of frailty and this likely helps to decrease the prevalence of depression (Chen et al., 2017). Thus, it can be said that frailty is a risk factor for depression among the older population in the community. Focusing on improving these risk factors for frailty will likely contribute to the reduction of depression in older adults.

While a negative relationship between economic income and depression was found by a previous study (Han et al., 2017), no correlation between economic income and depression was observed in the current study, which could be related to the bias of the selected samples. The participants in the sample population selected in this study are from the urban-rural areas of Wuhan and those who had a low-level monthly income in the city. However, due to the local urbanization, housing transformation has been undertaken by the government for the area in which the participants live, and each family has received 2–3 rounds of housing compensation and a certain amount of economic compensation. Consequently, their fixed and invisible assets are not low and there is no economic anxiety for them. Therefore, their monthly income does not fully reflect their actual economic status.

Previous evidence suggested a reciprocal relationship between depression and cognitive dysfunction. Recurrent major depression was a risk factor for Alzheimer’s disease, also Alzheimer’s disease constituted a risk factor for depression (Aznar and Knudsen, 2011). Reports have revealed that similar changes in the neuroplasticity, morphology, and neurotransmission in the brain seemed to be associated to depression and cognitive dysfunction (Hussain et al., 2020). Alzheimer’s disease intrinsically deteriorates affective regulation, a critical capacity in the resistance to depression in the face of life stresses (Nash et al., 2007). Alzheimer’s disease also promotes pro-inflammatory signaling and inhibits neuroplasticity, which has critical links to mood regulation (McEwen, 2004; Réus et al., 2016). In this study, we found cognitive dysfunction was correlated with depression. It suggested that interventions improving cognition would be a way to relieve depression.

The current study has a few limitations. The participants in this study were from four communities in the suburban areas of Wuhan, which may have led to selection bias. Moreover, because this was a cross-sectional study, prospective studies are required in order to validate the findings. In addition, we did not consider the associations of dietary habits, mental conditions, chronic pain, and multimorbidities with depression. Furthermore, a methodological improvement like assessing cognitive dysfunction in a more specific and sensitive manner must be made in future research. It will clarify the relationship between different aspects and degrees of cognitive function impairment and depression.

## Conclusion

Since geriatric depression has become a major public health concern, it is crucial to have measures such as early identification, prevention, and intervention management of geriatric depression in the community from a multidimensional perspective. In particular, strengthening social support and improving frailty in older adults must be encouraged to enable older adults to have optimal physical and mental health.

## Data Availability Statement

The raw data supporting the conclusions of this article will be made available by the authors, without undue reservation.

## Ethics Statement

The studies involving human participants were reviewed and approved by the Ethics Committee of Union Hospital, Tongji Medical College, Huazhong University of Science and Technology. The participants provided their written informed consent to participate in this study.

## Author Contributions

PH and ZHW designed study. AHL and PH wrote the manuscript. PH and YJP performed the statistical analysis. YJP, WLZ, YLZ, SHG, YZ, and KMZ collected and analyzed the data. All authors approved of the final version of this manuscript.

## Conflict of Interest

The authors declare that the research was conducted in the absence of any commercial or financial relationships that could be construed as a potential conflict of interest.

## Publisher’s Note

All claims expressed in this article are solely those of the authors and do not necessarily represent those of their affiliated organizations, or those of the publisher, the editors and the reviewers. Any product that may be evaluated in this article, or claim that may be made by its manufacturer, is not guaranteed or endorsed by the publisher.
